# Chemical Composition and Anti-*Candida* Activity of *Mentha suaveolens* Ehrh. Essential Oils Obtained by Different Distillation Processes

**DOI:** 10.3390/molecules28196934

**Published:** 2023-10-04

**Authors:** Vanja Tadić, Mijat Božović, Filippo Sapienza, Roberta Astolfi, Milan Mladenović, Maria Cristina Zaka, Fabiana Del Bove, Francesca Borzacchi, Caterina Fraschetti, Caterina Rossi, Silvia Vertuani, Anna Baldisserotto, Stefano Manfredini, Rino Ragno

**Affiliations:** 1 Institute of Medicinal Plants Research Dr. Josif Pančić, Tadeuša Koščuška 1, 11000 Belgrade, Serbia; vtadic@mocbilja.rs; 2 Faculty of Natural Science and Mathematics, University of Montenegro, Džordža Vašingtona bb, 81000 Podgorica, Montenegro; mijatboz@ucg.ac.me; 3 Rome Center for Molecular Design, Department of Drug Chemistry and Technology, Sapienza University of Rome, Piazzale Aldo Moro 5, 00185 Rome, Italy; filippo.sapienza@uniroma1.it (F.S.); roberta.astolfi@uniroma1.it (R.A.); 4 Kragujevac Center for Computational Biochemistry, Department of Chemistry, Faculty of Science, University of Kragujevac, Radoja Domanovića 12, 34000 Kragujevac, Serbia; milan.mladenovic@pmf.kg.ac.rs; 5 Department of Drug Chemistry and Technology, Bachelor Course in Applied Pharmaceutical Sciences, Sapienza University of Rome, Piazzale Aldo Moro 5, 00185 Rome, Italy; mariacristina.zaka@virgilio.it (M.C.Z.); fdelbove@gmail.com (F.D.B.); 6 Garden Center “98.3 Piante Mediterranee”, 01016 Tarquinia, Italy; francesca.borzacchi@gmail.com; 7 Department of Drug Chemistry and Technology, Sapienza University of Rome, Piazzale Aldo Moro 5, 00185 Rome, Italy; caterina.fraschetti@uniroma1.it; 8 Department of Life Sciences and Biotechnology, University of Ferrara, Via L. Borsari 46, 44121 Ferrara, Italy; caterina.rossi@unife.it (C.R.); vrs@unife.it (S.V.); bldnna@unife.it (A.B.)

**Keywords:** *Mentha suaveolens* Ehrh., 24-hour extraction, essential oils, distillation, piperitenone oxide, anti-*Candida* activity

## Abstract

A comparative study on essential oils extracted from *Mentha suaveolens* Ehrh. from Italy is reported. Two extraction procedures were investigated: hydrodistillation and steam distillation, carried out as a continuous and fractionated procedure. Fresh and dried plant material from two harvests was used. The hydrodistillation method yielded a higher amount of essential oil. The dried plant was significantly richer in essential oil per kg of starting plant material. Gas chromatography-mass spectrometry analysis of 112 samples showed that the essential oils belong to the piperitenone oxide-rich chemotype. In addition, piperitenone, p-cymen-8-ol, and limonene were among the most abundant compounds in the different samples. A higher amount of piperitenone oxide was obtained by hydrodistillation, while steam distillation gave a higher percentage of piperitenone and limonene. The essential oils were characterized for their anti-*Candida albicans* activity; higher potency was observed for the samples rich in piperitenone oxide, with MIC values ranging from 0.39 to 0.78 mg·mL^−1^ (0.039% and 0.078% p/v). The results of this work provide a deep insight into the methodology of essential oil extraction and the associated chemical variability of *M. suaveolens* Ehrh. Some of the essential oils are potent against *C. albicans* and could be considered for potential use in therapy.

## 1. Introduction

Essential oils (EOs) are complex, volatile mixtures usually extracted from aromatic plants. These olfactory secondary metabolites accumulate in specific secretory structures such as ducts and trichomes. Numerous methods have been reported to extract EOs from plant sources, the most commonly used being distillation, pressure (citrus oils by cold pressing), or extraction with various organic solvents or supercritical carbon dioxide. The volatile mixtures obtained are usually liquid at room temperature, slightly soluble in water, and soluble in organic solvents. EOs are made up of many different chemical compounds that have specific chemical and physical properties that give the oils their distinctive chemical, physical, and biological characteristics [1]. However, it is known that the yield and chemical composition of EOs can be influenced by several factors. Variability is due to physiological status, such as the stage of development of plant organs, seasonal variations, and part of the plant material (e.g., leaves, flowers, or the whole aerial part) or secretory structures. Different environmental conditions, such as climatic and edaphic factors, can strongly influence these parameters. In addition, the extraction method has a significant influence on the yield and composition of EOs; therefore, the exploration of extraction methods and their comparison in terms of quality and/or quantity of EO is an important challenge of applied research on EOs.

Among the methods used for essential oil (EO) production, distillation-based processes such as hydrodistillation (HD) and steam distillation (SD) are the most commonly used, either on a laboratory or industrial scale. These methods are preferred because of their simplicity and low economic investment requirements [2]. They both use heat to evaporate the EO, which is carried out of the plant by the steam produced, and then differences in density allow the condensed water and EO to be easily separated physically. In HD, the plant material is completely immersed in boiling water, whereas in SD, the plant material is kept separate, allowing the steam to enter and pass through. The temperature of the steam entering the plants can be different, being 100 °C for HD and higher for SD in the case of pressurized steam production. These processes are flexible, versatile, operate from small to large plant volumes, and generally do not lead to EO decomposition [2]. Disadvantages include losses of some volatile components and degradation of some unsaturated compounds by thermal effects or hydrolysis, long extraction times, and high energy consumption [3,4]. Therefore, recent research approaches aim at optimizing and improving existing techniques or introducing some new environmentally friendly methods, such as ultrasound and microwave-assisted processes. An inappropriate extraction procedure can lead to changes in the chemical signature of EOs, which is likely to result in artifacts with changes in bioactivity or organoleptic properties (e.g., color, odor, and/or flavor changes/losses) [5].

The effect of different distillation methods on EO content and chemical composition has been reported previously [6,7]. This aspect is of particular interest in terms of commercial-scale EO production and economic profit [8]. Furthermore, the effect of extraction time on these parameters has been studied in depth [9,10,11,12,13,14,15]. It was concluded that a longer distillation would result in a more complete EO composition but could lead to some artifacts and thus changes that may affect both the physical properties and biological activities of the EO [4]. This concept was investigated and led to the development of a 24-hour extraction model, which was first applied to *Mentha suaveolens* Ehrh. (MS). This aromatic herbaceous perennial plant belongs to the Lamiaceae family and has been used in traditional medicine in Mediterranean areas. EO from MS (MSEO) has been the subject of numerous studies that have shown a difference in its constituents, mainly depending on the region of origin [16,17,18,19]. In general, studies on the chemical composition of MSEO from different regions showed a high percentage of oxides [20]. The analysis of EO from wild MS collected in Tarquinia (Viterbo, Italy) showed a predominance of piperitenone oxide (PO) up to more than 90% [21]. However, fractionated and extended distillation showed large variations in PO yield depending on the separation interval and harvest period [14]. As a continuation of these studies, HD and SD were applied to MS harvests, and the procedures included both continuous (c-HD or c-SD) and fractionated extractions (f-HD or f-SD) from either fresh or dried plant material. The chemical compositions of the EO samples obtained are presented here, with particular emphasis on the variation in the content of major compounds. In order to relate the different compositions to the biological effect, the present study included an analysis of the anti-*Candida* activities of the obtained EO samples using PO as one of the references.

## 2. Results

### 2.1. EO Extraction

MS aerial parts were subjected to HD and SD, and the extractions were performed as continuous (c-HD and c-SD) and fractionated (f-HD and f-SD) distillations. In this way, the differences between the distillation methods were monitored, as well as the yield variations between fresh (fc-HD, fc-SD, ff-HD, or ff-SD) and dried plant material (dc-HD, dc-SD, df-HD, or df-SD). Since these types of extractions are susceptible to various factors, all processes were performed in duplicate from two harvests separated by 10 days. 14 extractions were performed per harvest (see Materials and Methods), yielding a total of 112 EO samples (Table 1).

#### 2.1.1. Continuous HD and SD

The plant material was subjected to continued SD and HD of different durations: 1, 2, 3, 6, and 24 h. To monitor the rest of the extraction process (up to 24 h), these distillations included additional extractions of 23, 22, 21, and 18 h, respectively (Table 1). Yields calculated per weight of fresh/dried plant material are shown in Table 2.

#### 2.1.2. Fractionated HD and SD

EO fractions were spilled at interval times of 1, 2, 3, 6, and 24 h, and the yields were calculated per weight of fresh or dried plant material (Table 3).

### 2.2. EO Chemical Composition

**Figure 1:**
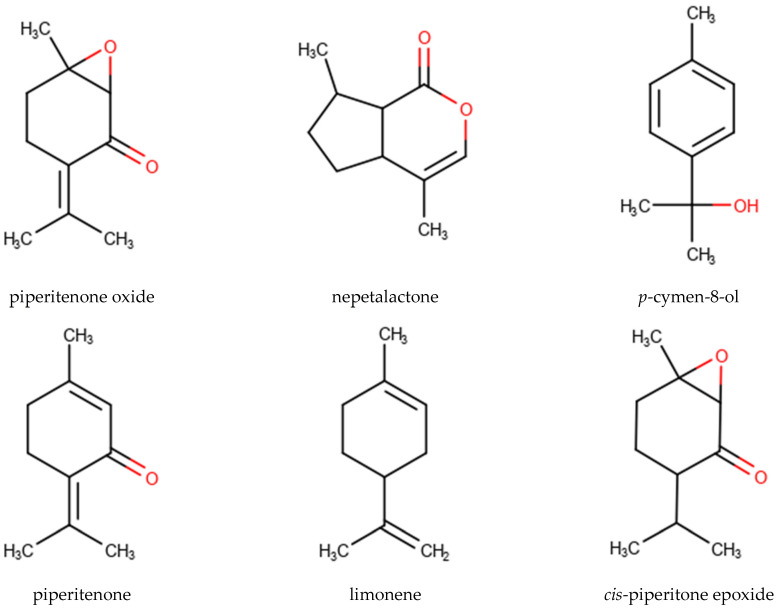
Chemical structure of the highest average percentages and top occurring compounds: piperitenone oxide (PO), nepetalactone (NPL), p-cymen-8-ol (PCY), piperitenone (PIP), limonene (LIM), and cis-piperitone epoxide (CPO).

A total of 112 EO samples were obtained by either fractionated or continuous SD and HD extractions (Table 1). Gas chromatography-mass spectrometry (GC-MS) analysis revealed the presence of 474 chemical constituents (Table S3) in different relative proportions in the different EOs. However, PO, piperitenone (PIP), nepetalactone (NPL), p-cymen-8-ol (PCY), limonene (LIM), and cis-piperitone epoxide (CPO) with the highest average percentages and peak abundances could be distinguished as the main characterizing components of the MSEO samples analyzed in this study (Figure 1, Table 4 and Table 5).

### 2.3. Anti-Candida Activity

The in vitro antifungal activity analysis included the majority of the samples, as some of them yielded very low amounts and it was not possible to investigate their antimicrobial efficacy. A total of 82 MSEO samples extracted by continuous and fractionated HD and SD were tested against Candida albicans (ATCC 10231), and the results are reported here (Table 6). The anti-Candida efficacy was compared with that of miconazole (minimum inhibitory concentration (MIC) = 0.016 mg·mL^−1^), a well-known synthetic antifungal drug, and with that of the solvent used to dilute the EOs as a blank (RPMI 1640 supplemented with Tween 80), which had no activity against C. albicans. The results presented here are representative of two independent experiments (24 and 48 h of incubation) performed in triplicate. The MIC of this strain ranged from 0.39 to 12.48 mg·mL^−1^. Notably, some of the samples showed interesting and potent antifungal activity, with MICs ranging from 0.39 to 0.78 mg·mL^−1^. The anti-Candida activity of isolated PO and synthesized PIP was also evaluated. The 24-hour MIC was 12.48 mg·mL^−1^ for synthesized PIP and 6.24 mg·mL^−1^ for PO. At 48 h, the MICs were 12.48 mg·mL^−1^ for both compounds.

## 3. Discussion

### 3.1. EO Extraction

It would be expected that prolonging the duration of the extraction process would lead to a slight increase in the cumulative amount of EO [22], especially considering the results obtained with the fractionated distillation process, which show significant yields up to 24 h (see Section 2.1.2). However, the results obtained in this study do not strictly follow this pattern, as the expected amount of EO may decrease with time extension. This aspect was not investigated further, as it could be due to several unknown reasons. The 24 h SD and HD extractions on fresh MS (first harvest) gave the lowest amounts of EOs, even more than three times less than the 6 h extractions. In the case of dried plant material, these exceptions are particularly noticeable in the 3-hour SD extractions. This is consistent with some literature data reporting a 25–40% decrease in yield for some *Cymbopogon* EOs with increasing time [23]. Numerous studies have confirmed that EO yield decreases with time [24,25,26] or at least reaches equilibrium at some point without further increase [27,28,29]. There are also data from the literature suggesting different effects of drying on EO content [30], as some studies noted a significant decrease in yield [31], while others reported an increase [26]. These differences could be due to the drying time as well as the temperature used [30].

From the results here, it can be observed that the second harvest yielded more EOs in both SD and HD processes, with only one exception in the 6 h SD from dried material (Supplementary Material, Figures S1 and S2). The results also show that drying MS significantly increases the EO content, similar to that reported for fractionated SD and HD (Supplementary Material, Figures S3 and S4). Comparing the two distillation methods applied, it seems that HD is more efficient since it gives a higher yield, especially in the case of the second harvest (Table 2 and Supplementary Material, Figures S5 and S6). As the two harvests were performed with a 10-day time difference and considering the high weather variability during September (harvest month), variability in both yield and composition could be expected.

The additional fractions from the continuous part up to 24 h and the cumulative yields (up to 24 h) were also examined (Table 2). Continuous 1-, 2-, 3-, and 6-hour distillations yielded from 14.42% to 49.39% of the total (24 h) yield (Supplementary Material, Table S1). With a few exceptions, it is also noticeable that MS drying increased these percentages, which is particularly evident in the case of HD extractions (up to two-fold). However, these data are not consistent with those obtained for fractionated distillations (see Section 2.1.2), where 6 h of extraction (first four fractions) were allowed to extract from 68.11% to 98.77% of the total EO amounts (Supplementary Material, Table S2).

Comparison of the same extraction types from two harvests revealed very small differences between the yield trends (Supplementary Material, Figures S7 and S8). In the case of fresh material subjected to SD, two main yield peaks were observed: the first between the first and second extraction hours, and the second between the third and sixth extraction hours. On the other hand, SD of the dried material yielded the highest amount of EO in the first hour of the extraction process, but with a remarkable addition in the last 18-hour fraction. In the case of HD, from 63.8% to 88.9% of the total amount of EO (extracted in 24 h) was isolated in the first 2 h (Supplementary Material, Table S2), which is common for many Lamiaceae species. However, there are large differences in yields between fresh and dried materials, as drying increased the total yield up to seven-fold in the case of SD and even 15-fold in the case of HD (Table 3 and Supplementary Material, Figures S9 and S10).

Comparing the two distillation methods, HD yielded a higher percentage of total EO extracted in the first 3 h of extraction (up to 91.9%), which is the most common extraction time (Supplementary Material, Table S2, and Figures S11 and S12 This is consistent with a previous report suggesting a 3-hour HD as the optimal duration for MSEO extraction [14].

### 3.2. EO Chemical Composition

As previously reported, MSEO from Tarquinia belongs to the PO-rich chemotype [14,21,32,33,34,35]. PO can certainly be highlighted as the main component, and it was found in all samples with relative percentages ranging from 1.2% (EO087) to 71.7% (EO068), and it is present in 25% of all EO samples with a percentage higher than 55.6% (Table 4 and Table 5). The results show that this monoterpenoid epoxyketone is usually extracted in the first 6 h of the extraction process, in agreement with a previous study [14]. This is particularly evident in the fractionated distillations: PO was the most abundant compound in the first four fractions, while its content was significantly reduced in the last 18 h of the extraction process. Examples include both SD and HD extractions, e.g., EO082, EO087, and EO107 (Table 4). PO was mainly extracted during the first 3 or 6 h of the extraction process, as shown by the results of the continued distillations. Consequently, the extractions performed to complete the 24-hour distillation processes provided EO samples with the lowest PO content. For example, sample EO049 obtained with a 6-hour continued HD extraction (2_fc-HD_0-6) contained 71.3% PO, while the corresponding additional 18-hour extraction (2_fc-HD_6-24) yielded only 3.2% PO (EO054).

It is also observed that the HD method gave a better yield of PO than the SD method, but only in the case of fractionated distillations. Moreover, it seems that the drying of the plant material did not have a significant effect on the PO content, although there were samples where the PO content even doubled, from EO079 with 27.3% in the second fraction (2_ff-SD_1-2) isolated from the fresh material by fractionated SD to EO089 with a PO content of 60.2% obtained from the dried plant with the same extraction method and the same fraction (2_df-SD_1-2).

Regardless of the fact that PO was the compound driving the MS chemotype, among all the EO samples, NPL showed the highest values, with a percentage up to 79.8%, as found in EO112, obtained by HD on dried plant in the last fraction of a fractionated distillation (2_df-HD_6-24). In fact, NPL was always abundant in all the EOs obtained in the longer distillation, with percentages starting at 4.30%. Correspondingly, in the first fractions, NPL was always present at low percentages or even absent in a few samples (EO019, EO020, EO057, EO078, EO079, and EO093). Nepetalactones are atypical monoterpenes of the iridoid group produced by some plants as defense compounds. The term itself includes several analogous stereoisomers. NPL has been found among the main constituents of these EOs. Its significant amount was usually reported after the first 2 h of extraction, reaching a maximum in the last 18-hour fraction: 16.4–79.8% and 4.9–27.7% in HD and SD, respectively. This is also evident in the EOs obtained by the continuous distillation processes: the NPL content increased with the prolongation of the extraction, thus significantly enriching the chemical composition of the samples obtained by long distillations. For example, 33.6% of NPL was reported in EO053 obtained by the 3–24 h HD extraction and 32.8% in sample EO017 obtained by the corresponding SD. There was no difference in yield between HD and SD. In addition, the drying of the plant material did not seem to have a significant effect on the accumulation of NPL. However, it is interesting to note that the EOs extracted from the plant material of the second harvest were significantly more abundant in NPL.

Along with glycosylated iridoids, nepetalactones are found in many Lamiaceae species, the most common source being *Nepeta cataria* L. (catnip), which is known for its feline-attracting properties [36]. These lactones are produced via a monoterpenoid pathway involving several oxidation processes on geraniol and a two-step enzyme-controlled cyclization leading to the formation of specific stereoisomers [36,37,38,39]. *Mentha* species are not considered to be an important source of nepetalactones; they are usually present in traces or low amounts in their EOs [40]. However, there are some reports of their significant content in *M. longifolia* (L.) Huds. [41,42] and even in MS [43,44]. On the other hand, several studies have shown a possible biosynthesis of nepetalactones from some typical mint monoterpene constituents, such as pulegone (PUL) [45,46], CPO [45], and LIM [47,48]. Considering the results presented here, it is possible that some transformations may occur due to the prolonged exposure to heat during the extended distillation process. In fact, LIM, for example, was mostly isolated during the first hours of extraction, while the NPL content became significant after this period.

As with PO, PCY and PIP were also found in each sample at concentrations ranging from 0.2% (EO011) to 47% (EO063) and 0.1% (EO098) to 32.9% (EO015), respectively. PCY appears to be randomly distributed across the EO samples, with a slight tendency to accumulate in the later fractions. Instead, a slightly different profile was observed for PIP, with this monoterpene ketone being characteristic of the latter fractions as its percentage reached a maximum after the first 3 h of extraction. This was particularly emphasized by the continuous distillations, in which the PIP content increased its percentage in the last 6 h, thus representing a chemical marker for long-lasting MS distillations. Interestingly, by comparing HD and SD, it could be excluded that SD allows a better extraction of PIP than HD, especially in the case of continuous SD, where its content was the highest. Regarding LIM and CPO, although they have a frequency of 69% and 75% (as shown in Table 5), their percentage reaches a maximum of 30.8% and 17.70%, with averages of 3.5% and 3.0%, respectively. Contrary to PCY and PIP, generally higher LIM concentrations were found in the first hour of distillation (30.8% as in 2_ff-SD_0-1), decreasing with increasing distillation time until disappearing in the last 18 h. A similar profile was also observed for CPO and its *trans*-geometric isomer (TPO), which were usually found in the first hours of extraction, particularly in the case of fractionated HD and SD, reaching up to 9.3% and 6.3%, respectively. Furthermore, in some samples from the continuous extractions, CPO and TPO participated to a large extent. For example, CPO was an important component in samples EO002, EO019, EO021, EO037, EO038, and EO039 (10.1–17.7%), and TPO reached a maximum of 20.1% in sample EO001. All of these samples were obtained by extractions lasting a maximum of 3 h. Continuous distillation processes showed that the content of PO and CPO (as epoxy forms) decreased with the extension of the extraction process, whereas the content of PIP increased. Considering the monoterpene biosynthetic pathway in *Mentha* species [49] and the very close relationships between the mentioned compounds, it is most likely that the longer extraction processes allowed different types of degradations or/and transformations, thus significantly changing the chemical outfits of these EOs.

The appearance of other constituents is related to the extraction time, but some of them are often present in significant amounts. A group of other monocyclic monoterpenes with a menthane skeleton should be mentioned here. LIM was often found in significant amounts in the EOs obtained by fractionating HD and SD. It was mostly isolated at the beginning of the extraction and significantly influenced the chemical composition of the first three fractions (up to 30.8%). The results led to the conclusion that SD was more potent in yielding LIM and that drying of the plant material seemed to enhance LIM accumulation. Interestingly, these findings were not supported by the results obtained with continuous distillation processes: LIM was not among the major compounds, although it was present in many of these samples.

The longer HD and the presence of two unsaturated double bonds make the LIM structure suitable for transformation into its numerous derivatives [50]. LIM could be the ancestor of the group that exemplifies various oxygenated forms, such as the aforementioned PIP, PO, CPO, and TPO [51]. However, a group of terpinenes is also formed from the same LIM-transformed intermediates, including γ-terpinene, which is thought to be the precursor of the phenolic derivative thymol (TYM) [52,53]. Alternatively, the same carbocation can be quenched by a water attack, resulting in the formation of α-terpineol. The formation of a heterocyclic ring on this alcohol leads to its conversion to 1,8-cineole (*syn.* eucalyptol, EUC) [51]. Significant amounts of EUC and TYM were found in the samples obtained by fractionated distillations. EUC was particularly abundant in those obtained by SD (up to 14.8%), mainly related to the first hours of extraction. In contrast, higher amounts of TYM were reported for the EOs isolated by HD (up to 11.4%), usually appearing in the last hours of extraction. Significantly lower amounts of EUC and TYM were found in EOs obtained by continuous HD and SD methods: up to 6.3% and 6.5%, respectively.

Oxidative processes of p-cymene, a by-product of TYM biosynthesis, lead to the formation of p-cymenene (CYN) and p-cymen-8-ol (PCY), both of which were found to be important constituents of the EOs analyzed. CYN was extracted much better with the fractionated distillations, especially with SD. In some of these EO samples, its content reached its maximum, e.g., 12.3% (EO086) and 44% (EO087). PCY was present in each sample, and it is interesting that those EOs extracted from the plant material of the first harvest were much more abundant in this compound. Furthermore, drying of the material increased its content up to more than 10 times: e.g., 1.6% in the third fraction (EO075) isolated from the fresh material by fractionated SD, and 22.9% in the same fraction (EO085) but obtained from the dried material. This aromatic monoterpenoid was mainly extracted between the second and sixth hours of extraction, although some of the long-continued extractions gave samples very rich in it, e.g., 18th hour EO063 and 22nd hour EO061 with 47.8% and 38.9% of PCY, respectively.

Some compounds from the sesquiterpene group are worth mentioning since they appeared in a large number of EO samples: e.g., α-cadinol (up to 4.1% and 2.8% in the EOs obtained by continuous and fractionated extractions, respectively), *trans*-caryophyllene (up to 6.1% and 5.9% in the EOs obtained by continuous and fractionated extractions, respectively), and its oxide (up to 5.5% and 2.7% in the EOs obtained by continuous and fractionated extractions, respectively).

Regarding other EO components, some were found in significant concentrations only in certain samples. For example, shisofuran in sample EO036 (5.2%), phytol in sample EO018 (7.6%), *trans*-phytol acetate (6.8% and 7.3% in EO072 and EO043, respectively), and 6-hydroxy carvotanacetone (6.6% and 5.1% in EO027 and EO009, respectively). Interestingly, a further 2-hour SD of dried MS material from the first harvest gave sample EO020 an abundance of compounds not significantly present in other EO samples: *cis*-mercapto-p-menthan-3-one (16.5%), citronellyl propanoate (10.5%), camphene hydrate (7.2%), and *cis*-carvyl acetate (5.5%).

It is important to mention that various structural changes of thermally labile terpenes can occur during extraction. Moreover, this is an expected scenario considering that the whole distillation process was often prolonged, so hydrolytic and/or oxidative degradations and/or transformations may have occurred [54]. For example, six-membered rings containing one or two double bonds may undergo dehydrogenation to form an aromatic system; this transformation has been reported for LIM and α-terpinene [55]. As a result, p-cymene, CYN, or TYM may be formed. Another possible modification is the formation of epoxides; e.g., it has been reported that α-terpinene can be converted to EUC. In fact, the results presented here showed the opposite evolution of LIM and some other compounds that could be considered as its degradation products, such as the TYM of CYN: the former decreased and the latter increased with the extraction time. Furthermore, the lactone structures are expected to degrade easily under prolonged distillation conditions. As bicyclic monounsaturated terpenoids, nepetalactones undergo primary oxidation to an unstable product and are then converted to stable secondary oxidation products such as alcohols, aldehydes, ketones, epoxides, or acids. Heat and light have been found to promote the cleavage of the unique double bond in NPL by epoxidation or allylic oxidation to alcohols, ketones, and aldehydes [56]. All of these possible changes should be considered, as the EOs analyzed showed great chemical variability depending on both the type and duration of extraction.

### 3.3. Anti-Candida Activity

The antimicrobial potential of MSEO has been thoroughly analyzed; there is a large amount of data in the literature, summarized in a previously published review [20]. Considering the EO chemistry of this plant depending on its origin, a large variability in its antimicrobial efficacy has been reported. For example, strong activity was observed for PUL-rich MSEO, while those rich in PO and/or piperitone oxide showed much weaker activity [57]. The importance of the chemical structure of several monoterpenoids for the expression of biocidal activity was investigated, most of which are common components of MSEO. The results obtained showed that LIM, carvone, and menthone were significantly less potent than PO, piperitone oxide, and PUL [57]. The authors concluded that the presence of an additional double bond in the molecules of PUL and PO (compared to the others) may be responsible for the higher potency.

Numerous studies have reported good or excellent antifungal activity of MSEO [33,35,58,59,60]. A previous analysis of the EOs extracted from the material collected in Tarquinia (Italy) showed strong anti-*Candida* activity for the samples rich in PO [14]. However, excellent potency was also reported for the sample containing 5.61% PO, but it was abundant in other compounds such as CYN (26.64%) and cinerolone (18.96%). Therefore, this study confirmed the phytocomplex hypothesis reported in many other experimental observations, according to which the overall expression of activity may result from synergistic and/or antagonistic mechanisms and cooperative interactions between different constituents [61]. Following this study, a further 82 EOs were tested, as presented here. The results obtained confirmed that PO can be considered the main active ingredient since the most effective EOs contained large amounts of it, e.g., EO032, EO037, EO064, EO065, EO108, and EO109 (51.3–71.1% of PO). However, the role of PO was found to be controversial considering that low antifungal potency was observed with pure PO. The results presented here are in good agreement with a previous study that demonstrated potent candidastatic and candidacidal activities of MSEO rich in PO in an in vitro experimental system [33]. Some other authors also attributed the strong antifungal activity of various plant EOs to the high content of PO [21,62,63,64].

In addition, samples EO035, EO070, and EO072 with average PO content (11.9–38.6%) also showed good activities. These were abundant in NPL (18.1–27.2%), which can be considered to enhance the efficacy of PO. In support of this, some other EO samples characterized by low PO content but rich in NPL lacked any significant activity (e.g., EO077, EO083, EO092, and EO102). As mentioned above, nepetalactones are the most dominant constituents in the EOs of different *Nepeta* species, often accounting for more than 80%. Several studies have demonstrated the high potency of these EOs against *Candida* strains [65,66,67,68,69]. However, the antimicrobial performance of these EOs and/or nepetalactones, known for their stereoisomeric diversity and associated variability in the expression of biological activities, may be difficult to predict.

Another important finding is the discovery of a cooperative interaction between the main components, which is also evident in some samples containing significant amounts of PIP, LIM, EUC, and PCY. For example, samples EO023 and EO024 showed good activity, most likely due to the large amounts of PO together with PIP (10.1% and 15.1%, respectively) and PCY (12.8% and 16.2%, respectively). The same was reported for samples EO073 and EO098, which were rich in LIM (15.3% and 10.9%, respectively) and EUC (12.8% and 14.7%, respectively) in addition to PO. Some other *Mentha* species are also rich in PIP, and authors have often speculated about the importance of this monoterpenoid in exerting the potent anti-*Candida* activity reported for their EOs [62,70,71]. Although usually not abundant, some EOs rich in PCY showed good antifungal potential [72,73]. Nevertheless, other authors reported a lack of anti-*Candida* activity [10,74]. On the other hand, as a common EO ingredient, LIM has been the subject of numerous studies, including those on its efficacy against *Candida* [75,76]. Studies report that LIM inhibits *C. albicans* growth by disrupting the cell membrane. It induces oxidative stress, leads to DNA damage, and results in cell cycle modulation and the induction of apoptosis [77]. Furthermore, some authors have reported the enormous potential of LIM in the treatment of invasive candidiasis due to its ability to inhibit adhesion and biofilm formation [78]. A review of the literature has revealed a wealth of data regarding the antifungal activity of EUC, including that against various *Candida* strains. Low to moderate efficacy is usually reported [79,80,81,82,83], although many authors suggest its synergistic activity with other EO components such as camphor [84,85]. Finally, the possibility of antagonistic effects should not be underestimated, as there are examples where large amounts of the mentioned monoterpenes did not induce any significant antifungal activity. For example, samples EO014 and EO015 were rich in PO (33.1% and 24.8%, respectively) and PIP (21.4% and 32.9%, respectively), whereas samples EO074, EO075, EO078, and EO079 were dominated by PO (23.8–29.1%) and LIM (25.5–30.8%). The authors believe that these data are important for further studies on the design of specific EO mixtures.

## 4. Materials and Methods

### 4.1. Plant Material

Fresh MS material was provided by the garden center “98.3 Piante Mediterranee” in Tarquinia (province of Viterbo, Italy). Aerial parts were collected in two harvests in September 2017 from wild MS present on the company’s property. For comparison between fresh and dried starting material, a part of the plants was dried in a shaded room for 21 days. The taxonomic identification of the species was carried out according to the official European and Italian flora [86,87]. Voucher specimens were deposited at the Department of Pharmaceutical Chemistry and Technology of the Sapienza University of Rome.

### 4.2. Extraction Methods

EO extraction was carried out using a Clevenger-type apparatus; specifically, MS plant materials were subjected to distillation using a steel apparatus (Albrigi Luigi E0131, Verona, Italy), and EO spilling was carried out at continuous or fractionated times (Table 1 and Table 7). The accumulated oil/water phases were extracted three times with diethyl ether (15 mL), then the organic layers were dried on anhydrous Na_2_SO_4_, filtered, and stripped of solvent under atmospheric conditions to obtain dried EOs. The two harvests allowed all extractions to be performed in duplicate. The process was carried out with either fresh or dried material to allow comparison between the EOs obtained. The prepared EOs were stored in tightly sealed dark vials at −18 °C until further analysis.

#### 4.2.1. Fractionated Distillation

Similar to what was previously reported [4], samples were taken at intervals of 1, 2, 3, 6, and 24 h during distillation. As a result, five fractions were obtained, of which three were of equal duration (1 h each), the fourth at 3 h, and the last at 18 h (Table 7).

#### 4.2.2. Continuous Distillation

In parallel with the fractionated extractions, five further continuous distillations of 1, 2, 3, 6, and 24 h were performed. These extractions also included subsequent complementary fractions for a total distillation time of 24 h, resulting in four additional EOs of 23, 22, 21, and 18 h, respectively (Table 7).

### 4.3. Preparation of Pure PO and PIP

Since both PO and PIP were not commercially available, they were isolated or synthesized. PO was isolated from MSEO as previously described [15]. PIP was obtained by the method described by Bergmann and Bracha [88]. The purity of PO and PIP (>98%) was verified by gas chromatography (GC) and H-NMR.

### 4.4. EO Chemical Analysis

The chemical composition of EOs was investigated by GC. The analysis was performed on an HP-5890 Series II GC apparatus (Hewlett-Packard, Waldbronn, Germany) equipped with a split-splitless injector and an automatic liquid sampler attached to an HP-5 column (25 m × 0.32 mm, layer thickness 0.52 μm) and a flame ionization detector (FID). The carrier gas (H_2_) flow rate was 1 mL/min, split ratio 1:30, injector temperature was 250 °C, detector temperature was 300 °C, while the column temperature was linearly programmed from 40 to 260 °C (at a rate of 4 °C/min) and then held isothermally at 260 °C for 10 min. Sample solutions (23.9 mg·mL^−1^) of dichloromethane were injected sequentially in the amount of 1 μL. The area percentage reports obtained as a result of processing the chromatograms were used as the basis for quantification analysis.

Gas chromatography-mass spectrometry (GC-MS) analysis was performed using the same analytical conditions as for GC-FID, together with a column HP-5MS (30 m × 0.25 mm, film thickness 0.25 μm) on an HP G 1800C series IIGCD system (Hewlett-Packard, Palo Alto, CA, USA). Helium (He) was used as the carrier gas. The transfer line was heated at 260 °C; mass spectra were acquired in electron ionization (EI) mode (70 eV) in the 40–450 m/z range. A 0.2 μL sample solution (23.9 mg·mL^−1^ dichloromethane) was injected. The components of the EOs were identified by comparing their mass spectra with those from the Wiley 275 and National Institute of Standards and Technology/National Bureau of Standards (NIST/NBS) libraries using various search engines. The identification of the compounds was achieved by comparing their retention indices and mass spectra with those found in the literature [89] and supplemented by the Automated Mass Spectral Deconvolution and Identification System software (AMDIS ver. 2.1, manufacturer, National Institute of Standards and Technology (NIST), Standard Reference Data Program, Gaithersburg, MD, USA), GC-MS libraries. Experimental retention index values were determined using calibrated Automated Mass Spectral Deconvolution and Identification System software (AMDIS ver. 2.1, National Institute of Standards and Technology (NIST), Standard Reference Data Program, Gaithersburg, MD, USA), GC-MS Libraries, compared to those reported in the available literature [89], and used as an additional tool to confirm the MS results. The relative proportion of EO components was expressed as percentages obtained by peak area normalization, with all relative response factors taken as one.

### 4.5. Antimicrobial Activity Assay

The minimum inhibitory concentration (MIC) was determined by the microbroth dilution method (microsterile plate) according to the Clinical and Laboratory Standards Institute/National Committee for Clinical Laboratory Standards, as previously reported [4,10,11,12,13,14,90]. Miconazole (0.5 mg·mL^−1^), used as a positive control, was prepared by dissolving the drug in endotoxin-free water. EO solutions (124 mg·mL^−1^) were prepared in RPMI 1640 supplemented with Tween 80; the same was true for samples of PIP and PO. Briefly, to determine the MIC of MSEO samples extracted at different times (extraction times) and by different distillation methods or pure compounds (miconazole, PO, or PIP), RPMI-1640 supplemented with MOPS at pH 7 was used. EO was diluted in RPMI-1640 supplemented with Tween 80 (final concentration of 0.001% v/v). Dilutions of EO, PO, and PIP in 10 increasing concentrations ranging from 0.0244 to 12.48 mg·mL^−1^ were prepared in 96-well plates. The inoculum size was approximately 2.5 × 10^3^ cells·mL^−1^. The plates were incubated at 37 °C for 24 and 48 h. PO and PIP were also used as additional controls.

## 5. Conclusions

As a continuation of a previously reported study [14] and as part of a long-running investigation on MS, a detailed extraction procedure for MSEO using two distillation methods (HD and SD), both performed as continuous and fractionated, is presented here. Fresh and dried plant material from two harvests was used. According to the results obtained, the HD method can be considered superior in terms of EO yield. Drying of the plant material significantly increased the EO content, which was particularly evident in the case of HD extractions.

GC-MS analysis of the EO samples revealed the presence of numerous chemical constituents, with PO, NPL, PCY, PIP, LIM, and CPO being the most dominant. PO, as the main chemotype-determining compound, was usually isolated within the first 3–6 h of extraction, whereas PIP was characteristic of the later fractions or the longer continuous extractions. LIM significantly affected the chemical composition of the first three fractions, while PCY was mainly isolated between the second and sixth hours of extraction. In contrast, NPL was usually reported after the first 2 h of extraction. As observed, the HD method gave a better PO yield, but the drying of the plant did not significantly affect its content. On the other hand, the SD method allowed for better extraction of PIP and LIM than HD.

The study included the analysis of anti-*Candida* efficacy; a strong activity was observed for the PO-rich samples (51.3–71.1%), with the MIC value ranging from 0.39 to 0.78 mg·mL^−1^. Therefore, PO can be considered the main active ingredient, in good agreement with previous findings [14,21,33]. However, there are examples where the lower PO concentrations caused excellent activity, most likely due to their synergistic effects with PIP, LIM, and NPL. Furthermore, significant amounts of PCY and EUC in some of the samples could be involved in the expression of antifungal activity. The discovery of synergistic and antagonistic effects also opens a window for the possible preparation of specifically designed mixtures. An EO could be considered a kind of combinatorial library of small molecules, the composition of which also depends on the extraction method. In view of the increasing phenomenon of resistance to traditional antimicrobial agents, the characterization of the component and the correlation with the activity represent an important tool in the discovery of cheap antimicrobial agents. Moreover, the complexity of the mixtures, with the advent of increasingly powerful artificial intelligence systems, is no longer a problem, conferring on these “traditional” remedies a newer relevant source of active molecules with respect to biodiversity and an emphasis on sustainable approaches.

The results of this work provide deeper insights into the EO extraction methodology and the related chemical variability of MS with the origin of Tarquinia in Italy. Some EO samples are very potent against *C. albicans,* and they could be considered for their possible application in therapy. This is of particular interest since *C. albicans* is one of the most important causes of opportunistic infections worldwide, mostly affecting immunocompromised or hospitalized patients [91,92]. In vitro studies cannot be directly extrapolated to in vivo effects; therefore, the use of MSEO should be further investigated. In the case of in vivo use in clinical practice, the potential intolerance and toxicity of some of the components should be considered.

## Figures and Tables

**Table 1:** The complete list of EOs with ID, name, harvesting number, and plant state description.

	EO ID	EO Name ^1^	Harvest	Plant	EO ID	EO Name	Harvest	Plant
**Continuous Steam Distillation**	EO001	1_fc-SD_0-1	1	Samples from fresh plant material	EO019	1_dc-SD_0-1	1	Samples from dried plant material.
	EO002	1_fc-SD_0-2			EO020	1_dc-SD_0-2		
	EO003	1_fc-SD_0-3			EO021	1_dc-SD_0-3		
	EO004	1_fc-SD_0-6			EO022	1_dc-SD_0-6		
	EO005	1_fc-SD_0-24			EO023	1_dc-SD_0-24		
	EO006	1_fc-SD_1-24			EO024	1_dc-SD_1-24		
	EO007	1_fc-SD_2-24			EO025	1_dc-SD_2-24		
	EO008	1_fc-SD_3-24			EO026	1_dc-SD_3-24		
	EO009	1_fc-SD_6-24			EO027	1_dc-SD_6-24		
	EO010	2_fc-SD_0-1	2		EO028	2_dc-SD_0-1	2	
	EO011	2_fc-SD_0-2			EO029	2_dc-SD_0-2		
	EO012	2_fc-SD_0-3			EO030	2_dc-SD_0-3		
	EO013	2_fc-SD_0-6			EO031	2_dc-SD_0-6		
	EO014	2_fc-SD_0-24			EO032	2_dc-SD_0-24		
	EO015	2_fc-SD_1-24			EO033	2_dc-SD_1-24		
	EO016	2_fc-SD_2-24			EO034	2_dc-SD_2-24		
	EO017	2_fc-SD_3-24			EO035	2_dc-SD_3-24		
	EO018	2_fc-SD_6-24			EO036	2_dc-SD_6-24		
**Continuous Hydrodistillation**	EO037	1_fc-HD_0-1	1	Samples from fresh plant material	EO055	1_dc-HD_0-1	1	Samples from dried plant material.
	EO038	1_fc-HD_0-2			EO056	1_dc-HD_0-2		
	EO039	1_fc-HD_0-3			EO057	1_dc-HD_0-3		
	EO040	1_fc-HD_0-6			EO058	1_dc-HD_0-6		
	EO041	1_fc-HD_0-24			EO059	1_dc-HD_0-24		
	EO042	1_fc-HD_1-24			EO060	1_dc-HD_1-24		
	EO043	1_fc-HD_2-24			EO061	1_dc-HD_2-24		
	EO044	1_fc-HD_3-24			EO062	1_dc-HD_3-24		
	EO045	1_fc-HD_6-24			EO063	1_dc-HD_6-24		
	EO046	2_fc-HD_0-1	2		EO064	2_dc-HD_0-1	2	
	EO047	2_fc-HD_0-2			EO065	2_dc-HD_0-2		
	EO048	2_fc-HD_0-3			EO066	2_dc-HD_0-3		
	EO049	2_fc-HD_0-6			EO067	2_dc-HD_0-6		
	EO050	2_fc-HD_0-24			EO068	2_dc-HD_0-24		
	EO051	2_fc-HD_1-24			EO069	2_dc-HD_1-24		
	EO052	2_fc-HD_2-24			EO070	2_dc-HD_2-24		
	EO053	2_fc-HD_3-24			EO071	2_dc-HD_3-24		
	EO054	2_fc-HD_6-24			EO072	2_dc-HD_6-24		
**Fractionated Stem Distillation**	EO073	1_ff-SD_0-1	1	Samples from fresh plant material.	EO083	1_df-SD_0-1	1	Samples from dried plant material.
	EO074	1_ff-SD_1-2			EO084	1_df-SD_1-2		
	EO075	1_ff-SD_2-3			EO085	1_df-SD_2-3		
	EO076	1_ff-SD_3-6			EO086	1_df-SD_3-6		
	EO077	1_ff-SD_6-24			EO087	1_df-SD_6-24		
	EO078	2_ff-SD_0-1	2		EO088	2_df-SD_0-1	2	
	EO079	2_ff-SD_1-2			EO089	2_df-SD_1-2		
	EO080	2_ff-SD_2-3			EO090	2_df-SD_2-3		
	EO081	2_ff-SD_3-6			EO091	2_df-SD_3-6		
	EO082	2_ff-SD_6-24			EO092	2_df-SD_6-24		
**Fractionated Hydrodistillation**	EO093	1_ff-HD_0-1	1	Samples from fresh plant material.	EO103	1_df-HD_0-1	1	Samples from dried plant material.
	EO094	1_ff-HD_1-2			EO104	1_df-HD_1-2		
	EO095	1_ff-HD_2-3			EO105	1_df-HD_2-3		
	EO096	1_ff-HD_3-6			EO106	1_df-HD_3-6		
	EO097	1_ff-HD_6-24			EO107	1_df-HD_6-24		
	EO098	2_ff-HD_0-1	2		EO108	2_df-HD_0-1	2	
	EO099	2_ff-HD_1-2			EO109	2_df-HD_1-2		
	EO100	2_ff-HD_2-3			EO110	2_df-HD_2-3		
	EO101	2_ff-HD_3-6			EO111	2_df-HD_3-6		
	EO102	2_ff-HD_6-24			EO112	2_df-HD_6-24		

^1^ The EO name was composed with a number indicating the first or second harvest, then the type of distillation (HD or SD) was preceded by two letters, the first indicating the plant state (f for fresh and d for dry), the second indicating the distillation methodology (c for continuous and f for fractionated), and the last indicating the time or fraction of distillation in hours from a total of 24.

**Table 2:** EOs yields % per weight of fresh/dried plant material for each extraction from continuous distillations.

EO ID	EO Name ^1^	Yield %		EO ID	EO Name	Yield %	
		Single ^2^	Sum ^3^			Single	Sum
**Steam Distillation**							
EO001	1_fc-SD_0-1	0.0161	-	EO019	1_dc-SD_0-1	0.4217	-
EO002	1_fc-SD_0-2	0.0854	-	EO020	1_dc-SD_0-2	0.3844	-
EO003	1_fc-SD_0-3	0.0774	-	EO021	1_dc-SD_0-3	0.1791	-
EO004	1_fc-SD_0-6	0.1037	-	EO022	1_dc-SD_0-6	0.4881	-
EO005	1_fc-SD_0-24	0.0298	-	EO023	1_dc-SD_0-24	0.4211	-
EO006	1_fc-SD_1-24	0.0286	0.0447	EO024	1_dc-SD_1-24	0.2483	0.6700
EO007	1_fc-SD_2-24	0.1522	0.2376	EO025	1_dc-SD_2-24	0.3042	0.6886
EO008	1_fc-SD_3-24	0.0316	0.1090	EO026	1_dc-SD_3-24	0.2694	0.4485
EO009	1_fc-SD_6-24	0.1037	0.2074	EO027	1_dc-SD_6-24	0.0784	0.5665
EO010	2_fc-SD_0-1	0.0215	-	EO028	2_dc-SD_0-1	0.5057	-
EO011	2_fc-SD_0-2	0.0894	-	EO029	2_dc-SD_0-2	0.5477	-
EO012	2_fc-SD_0-3	0.0922	-	EO030	2_dc-SD_0-3	0.2517	-
EO013	2_fc-SD_0-6	0.1236	-	EO031	2_dc-SD_0-6	0.4019	-
EO014	2_fc-SD_0-24	0.1547	-	EO032	2_dc-SD_0-24	0.7939	-
EO015	2_fc-SD_1-24	0.0956	0.1171	EO033	2_dc-SD_1-24	0.3568	0.8625
EO016	2_fc-SD_2-24	0.0274	0.1168	EO034	2_dc-SD_2-24	0.3802	0.9279
EO017	2_fc-SD_3-24	0.0356	0.1278	EO035	2_dc-SD_3-24	0.4434	0.6951
EO018	2_fc-SD_6-24	0.0030	0.1266	EO036	2_dc-SD_6-24	0.1610	0.5629
**Hydrodistillation**							
EO037	1_fc-HD_0-1	0.0791	-	EO055	1_dc-HD_0-1	0.1431	-
EO038	1_fc-HD_0-2	0.0592	-	EO056	1_dc-HD_0-2	0.3806	-
EO039	1_fc-HD_0-3	0.1435	-	EO057	1_dc-HD_0-3	0.3001	-
EO040	1_fc-HD_0-6	0.1385	-	EO058	1_dc-HD_0-6	0.3903	-
EO041	1_fc-HD_0-24	0.0451	-	EO059	1_dc-HD_0-24	0.1563	-
EO042	1_fc-HD_1-24	0.0379	0.1170	EO060	1_dc-HD_1-24	0.1306	0.2737
EO043	1_fc-HD_2-24	0.0184	0.0776	EO061	1_dc-HD_2-24	0.1420	0.5226
EO044	1_fc-HD_3-24	0.0308	0.1743	EO062	1_dc-HD_3-24	0.0729	0.3730
EO045	1_fc-HD_6-24	0.0162	0.1547	EO063	1_dc-HD_6-24	0.0570	0.4473
EO046	2_fc-HD_0-1	0.1231	-	EO064	2_dc-HD_0-1	1.0613	-
EO047	2_fc-HD_0-2	0.2073	-	EO065	2_dc-HD_0-2	0.9434	-
EO048	2_fc-HD_0-3	0.1798	-	EO066	2_dc-HD_0-3	1.1995	-
EO049	2_fc-HD_0-6	0.1694	-	EO067	2_dc-HD_0-6	1.1056	-
EO050	2_fc-HD_0-24	0.1343	-	EO068	2_dc-HD_0-24	1.1174	-
EO051	2_fc-HD_1-24	0.0665	0.1896	EO069	2_dc-HD_1-24	0.2946	1.3559
EO052	2_fc-HD_2-24	0.0430	0.2503	EO070	2_dc-HD_2-24	0.3494	1.2928
EO053	2_fc-HD_3-24	0.0316	0.2114	EO071	2_dc-HD_3-24	0.1459	1.3454
EO054	2_fc-HD_6-24	0.0091	0.1785	EO072	2_dc-HD_6-24	0.0735	1.1791

^1^ The EO name was compiled as reported in Table 2; ^2^ Yield% of the single extract; ^3^ Cumulative yield% in the 24-hour extraction.

**Table 3:** EO yields% per weight of fresh/dried plant material for each fraction obtained from fractionated HD and SD.

EO ID	EO Name ^1^	Yield %		EO ID	EO Name	Yield %	
		Single ^2^	Sum ^3^			Single	Sum
**Steam Distillation**							
EO073	1_ff-SD_0-1	0.0197	0.0197	EO083	1_df-SD_0-1	0.2768	0.2768
EO074	1_ff-SD_1-2	0.0314	0.0511	EO084	1_df-SD_1-2	0.0380	0.3148
EO075	1_ff-SD_2-3	0.0187	0.0698	EO085	1_df-SD_2-3	0.0092	0.3240
EO076	1_ff-SD_3-6	0.0377	0.1075	EO086	1_df-SD_3-6	0.0527	0.3767
EO077	1_ff-SD_6-24	0.0174	0.1249	EO087	1_df-SD_6-24	0.0737	0.4504
EO078	2_ff-SD_0-1	0.0236	0.0236	EO088	2_df-SD_0-1	0.3073	0.3073
EO079	2_ff-SD_1-2	0.0271	0.0507	EO089	2_df-SD_1-2	0.0685	0.3758
EO080	2_ff-SD_2-3	0.0145	0.0652	EO090	2_df-SD_2-3	0.0818	0.4576
EO081	2_ff-SD_3-6	0.0233	0.0885	EO091	2_df-SD_3-6	0.1320	0.5896
EO082	2_ff-SD_6-24	0.0218	0.1103	EO092	2_df-SD_6-24	0.2761	0.8657
**Hydrodistillation**							
EO093	1_ff-HD_0-1	0.0283	0.0283	EO103	1_df-HD_0-1	0.6911	0.6911
EO094	1_ff-HD_1-2	0.0117	0.0400	EO104	1_df-HD_1-2	0.0458	0.7369
EO095	1_ff-HD_2-3	0.0053	0.0453	EO105	1_df-HD_2-3	0.0257	0.7626
EO096	1_ff-HD_3-6	0.0084	0.0537	EO106	1_df-HD_3-6	0.0195	0.7821
EO097	1_ff-HD_6-24	0.0007	0.0544	EO107	1_df-HD_6-24	0.0468	0.8289
EO098	2_ff-HD_0-1	0.0473	0.0473	EO108	2_df-HD_0-1	0.1086	0.1086
EO099	2_ff-HD_1-2	0.0228	0.0701	EO109	2_df-HD_1-2	0.0319	0.1405
EO100	2_ff-HD_2-3	0.0124	0.0825	EO110	2_df-HD_2-3	0.0093	0.1498
EO101	2_ff-HD_3-6	0.0111	0.0936	EO111	2_df-HD_3-6	0.0254	0.1752
EO102	2_ff-HD_6-24	0.0162	0.1098	EO112	2_df-HD_6-24	0.0203	0.1955

^1^ The EO name was compiled as reported in Table 1; ^2^ Yield% of the single extract; ^3^ Cumulative yield% in the 24 extraction hours.

**Table 4:** The relative percentages of the main compounds in each EO sample obtained.

EO ID ^1^	EO_Name ^2^	PO ^3^	NPL ^4^	PCY ^5^	PIP ^6^	LIM ^7^	CPO ^8^
EO001	1_fc-SD_0-1	55.30	1.60	1.90	2.90	0.00	3.70
EO002	1_fc-SD_0-2	45.90	0.40	1.80	6.20	1.40	17.70
EO003	1_fc-SD_0-3	50.90	0.50	4.80	2.70	1.60	9.20
EO004	1_fc-SD_0-6	59.80	2.40	2.70	2.20	1.50	7.50
EO005	1_fc-SD_0-24	22.60	16.50	6.40	18.90	0.20	1.10
EO006	1_fc-SD_1-24	33.10	12.40	6.60	16.10	0.30	0.90
EO007	1_fc-SD_2-24	38.80	7.00	7.20	8.70	2.10	5.80
EO008	1_fc-SD_3-24	27.10	14.90	9.80	12.40	0.00	0.60
EO009	1_fc-SD_6-24	51.80	8.30	5.70	5.10	0.10	4.20
EO010	2_fc-SD_0-1	27.80	2.90	1.70	7.10	0.10	4.40
EO011	2_fc-SD_0-2	60.50	3.10	0.20	2.10	0.10	0.40
EO012	2_fc-SD_0-3	57.10	2.70	1.20	9.50	1.70	0.10
EO013	2_fc-SD_0-6	68.20	0.40	1.30	4.70	1.90	0.60
EO014	2_fc-SD_0-24	33.10	9.80	1.10	21.40	3.40	0.20
EO015	2_fc-SD_1-24	24.80	10.10	1.30	32.90	0.10	0.10
EO016	2_fc-SD_2-24	55.10	7.10	1.70	11.90	0.10	0.00
EO017	2_fc-SD_3-24	9.00	32.80	2.40	18.40	0.10	0.00
EO018	2_fc-SD_6-24	17.20	15.90	9.30	11.20	0.00	0.00
EO019	1_dc-SD_0-1	16.90	0.00	7.50	1.30	14.60	10.20
EO020	1_dc-SD_0-2	12.70	0.00	11.60	5.20	0.90	6.90
EO021	1_dc-SD_0-3	15.10	0.30	18.30	2.30	4.10	10.10
EO022	1_dc-SD_0-6	32.40	0.30	12.90	4.10	6.70	6.80
EO023	1_dc-SD_0-24	32.60	5.00	12.80	10.10	0.40	4.70
EO024	1_dc-SD_1-24	28.40	7.10	16.20	15.10	0.00	1.30
EO025	1_dc-SD_2-24	20.20	10.50	17.10	13.20	0.60	0.10
EO026	1_dc-SD_3-24	14.50	14.10	17.10	8.10	0.50	0.30
EO027	1_dc-SD_6-24	7.90	12.80	24.80	9.80	0.00	0.60
EO028	2_dc-SD_0-1	65.10	0.80	0.90	1.10	0.90	2.10
EO029	2_dc-SD_0-2	60.60	0.20	1.80	5.30	2.00	2.10
EO030	2_dc-SD_0-3	53.40	0.20	2.20	9.20	1.30	1.60
EO031	2_dc-SD_0-6	43.60	6.00	1.90	16.60	0.90	1.10
EO032	2_dc-SD_0-24	51.30	5.10	1.20	19.20	0.00	0.60
EO033	2_dc-SD_1-24	50.50	14.00	1.80	9.90	0.00	0.00
EO034	2_dc-SD_2-24	23.10	27.10	1.80	13.30	0.00	0.00
EO035	2_dc-SD_3-24	14.70	27.20	1.60	19.70	0.00	0.00
EO036	2_dc-SD_6-24	6.60	31.20	1.90	22.40	0.00	0.00
EO037	1_fc-HD_0-1	56.90	0.50	2.50	0.80	0.60	12.80
EO038	1_fc-HD_0-2	55.20	0.30	2.20	0.70	0.80	12.30
EO039	1_fc-HD_0-3	56.40	0.90	1.70	0.80	1.40	10.80
EO040	1_fc-HD_0-6	56.80	1.70	1.80	0.80	2.60	9.00
EO041	1_fc-HD_0-24	30.40	6.80	9.10	1.20	1.20	3.70
EO042	1_fc-HD_1-24	33.90	8.40	15.10	3.30	0.00	1.20
EO043	1_fc-HD_2-24	3.20	22.80	17.50	6.10	0.00	0.00
EO044	1_fc-HD_3-24	59.30	8.20	6.80	2.90	0.00	0.90
EO045	1_fc-HD_6-24	62.50	7.10	7.20	3.10	0.00	2.60
EO046	2_fc-HD_0-1	56.90	0.50	2.50	0.80	0.60	12.80
EO047	2_fc-HD_0-2	55.20	0.30	2.20	0.70	0.80	12.30
EO048	2_fc-HD_0-3	56.40	0.90	1.70	0.80	1.40	10.80
EO049	2_fc-HD_0-6	56.80	1.70	1.80	0.80	2.60	9.00
EO050	2_fc-HD_0-24	30.40	6.80	9.10	1.20	1.20	3.70
EO051	2_fc-HD_1-24	33.90	8.40	15.10	3.30	0.00	1.20
EO052	2_fc-HD_2-24	3.20	22.80	17.50	6.10	0.00	0.00
EO053	2_fc-HD_3-24	59.30	8.20	6.80	2.90	0.00	0.90
EO054	2_fc-HD_6-24	62.50	7.10	7.20	3.10	0.00	2.60
EO055	1_dc-HD_0-1	43.90	0.60	6.70	0.70	5.30	9.90
EO056	1_dc-HD_0-2	47.70	0.20	6.20	0.70	6.80	9.80
EO057	1_dc-HD_0-3	48.00	0.00	6.20	0.70	6.50	9.90
EO058	1_dc-HD_0-6	45.80	0.30	10.40	1.10	2.80	9.80
EO059	1_dc-HD_0-24	38.40	5.80	13.30	1.90	1.20	6.80
EO060	1_dc-HD_1-24	28.10	10.60	22.50	2.30	0.00	1.20
EO061	1_dc-HD_2-24	12.80	8.50	38.90	2.30	0.00	0.30
EO062	1_dc-HD_3-24	10.20	13.60	24.50	3.10	0.00	0.30
EO063	1_dc-HD_6-24	3.10	12.40	47.80	3.10	0.00	0.50
EO064	2_dc-HD_0-1	71.70	0.90	0.60	0.30	1.50	1.00
EO065	2_dc-HD_0-2	58.20	0.70	0.90	1.10	3.80	3.70
EO066	2_dc-HD_0-3	61.90	0.70	0.80	1.20	3.30	2.50
EO067	2_dc-HD_0-6	67.10	0.40	1.20	1.50	1.00	2.20
EO068	2_dc-HD_0-24	71.70	4.30	1.20	2.00	0.10	0.70
EO069	2_dc-HD_1-24	48.80	13.90	2.80	4.70	0.00	0.00
EO070	2_dc-HD_2-24	38.60	18.10	2.80	3.60	0.00	0.00
EO071	2_dc-HD_3-24	12.10	26.50	6.10	8.00	0.00	0.00
EO072	2_dc-HD_6-24	11.90	21.20	7.10	6.60	0.00	0.00
EO073	1_ff-SD_0-1	36.50	0.20	1.70	0.50	15.30	2.90
EO074	1_ff-SD_1-2	29.10	0.10	1.20	0.30	26.70	1.50
EO075	1_ff-SD_2-3	28.00	0.20	1.60	0.50	25.50	1.00
EO076	1_ff-SD_3-6	29.50	6.50	2.50	1.20	11.60	0.70
EO077	1_ff-SD_6-24	15.10	20.70	4.20	3.10	3.70	0.30
EO078	2_ff-SD_0-1	23.80	0.00	0.40	1.10	30.80	0.20
EO079	2_ff-SD_1-2	27.30	0.00	0.60	2.30	27.70	0.00
EO080	2_ff-SD_2-3	43.30	0.20	1.40	6.20	9.40	0.10
EO081	2_ff-SD_3-6	31.00	8.70	1.20	9.40	3.60	0.00
EO082	2_ff-SD_6-24	3.10	22.30	2.10	6.50	1.00	0.00
EO083	1_df-SD_0-1	26.90	0.20	4.50	0.40	15.50	7.70
EO084	1_df-SD_1-2	36.90	0.40	17.40	1.70	1.20	3.80
EO085	1_df-SD_2-3	32.90	7.30	22.90	2.70	0.00	1.30
EO086	1_df-SD_3-6	11.50	6.20	20.60	2.40	1.20	1.40
EO087	1_df-SD_6-24	1.20	4.90	8.60	1.70	1.90	0.00
EO088	2_df-SD_0-1	32.10	0.10	0.70	0.50	24.10	1.10
EO089	2_df-SD_1-2	60.20	0.40	1.90	2.80	2.80	0.90
EO090	2_df-SD_2-3	51.30	6.20	3.40	4.00	1.20	0.40
EO091	2_df-SD_3-6	33.00	19.20	3.10	6.20	0.70	0.00
EO092	2_df-SD_6-24	8.40	27.70	3.20	7.30	1.20	0.00
EO093	1_ff-HD_0-1	24.50	0.00	0.90	0.30	17.80	7.20
EO094	1_ff-HD_1-2	45.10	0.30	3.00	0.80	6.90	4.70
EO095	1_ff-HD_2-3	57.30	2.70	5.30	1.60	0.10	2.00
EO096	1_ff-HD_3-6	28.80	8.40	5.60	1.90	2.60	0.90
EO097	1_ff-HD_6-24	32.90	16.40	9.00	3.00	0.00	0.00
EO098	2_ff-HD_0-1	42.30	1.00	0.60	0.10	10.90	0.30
EO099	2_ff-HD_1-2	64.60	0.70	0.90	2.80	5.50	0.00
EO100	2_ff-HD_2-3	70.70	2.00	0.90	4.10	3.90	0.00
EO101	2_ff-HD_3-6	60.40	8.90	1.40	5.80	1.60	0.00
EO102	2_ff-HD_6-24	5.00	36.20	2.70	7.20	1.30	0.00
EO103	1_df-HD_0-1	28.20	0.10	1.50	0.50	17.40	9.30
EO104	1_df-HD_1-2	36.30	0.70	4.80	0.80	14.90	7.30
EO105	1_df-HD_2-3	44.30	0.70	8.70	2.00	5.60	3.90
EO106	1_df-HD_3-6	26.10	13.00	8.70	2.40	0.00	1.30
EO107	1_df-HD_6-24	1.90	22.70	12.50	3.20	0.00	0.00
EO108	2_df-HD_0-1	53.90	0.30	0.80	1.00	8.00	1.40
EO109	2_df-HD_1-2	69.00	0.60	1.20	2.10	0.00	0.60
EO110	2_df-HD_2-3	59.30	10.20	1.40	3.30	0.00	0.00
EO111	2_df-HD_3-6	52.40	13.50	1.50	4.20	0.00	0.00
EO112	2_df-HD_6-24	5.10	79.80	1.50	2.70	0.00	0.00

^1^ Essential oil ID; ^2^ Sample names are composed as explained in Table 1; ^3^ PO = piperitenone oxide; ^4^ NPL = nepetalactone; ^5^ PCY = p-cymen-8-ol; ^6^ PIP = piperitenone; ^7^ LIM = limonene; ^8^ CPO = *cis*-piperitone epoxide.

**Table 5:** Top 30 most frequent chemical components across all the 112 MSEO samples, with details on their frequencies (%), average abundance, and variance. Data on percentiles are also included, along with minimum and maximum percentages.

Compound	Frequencies	Mean	STD	Min	Percentiles					Max
					25%	50%	75%	90%	95%	
piperitenone oxide	100.0	37.6	19.7	1.2	24.3	36.4	55.6	60.6	66.0	71.7
nepetalactone	94.6	8.2	11.0	0.0	0.5	5.5	12.4	22.2	27.1	79.8
p-cymen-8-ol	100.0	6.5	7.9	0.2	1.5	2.8	8.7	17.1	21.5	47.8
piperitenone	100.0	5.1	5.8	0.1	1.2	2.9	6.5	13.1	18.6	32.9
limonene	69.6	3.5	6.5	0.0	0.0	1.0	3.3	11.5	17.6	30.8
cis-piperitone epoxide	75.0	3.0	4.0	0.0	0.1	1.1	4.3	9.8	10.8	17.7
thymol	88.4	1.9	2.3	0.0	0.2	1.0	3.2	4.9	6.4	11.4
trans-caryophyllene	77.7	1.6	1.5	0.0	0.2	1.3	2.3	3.5	4.4	5.9
pseudodiosphenol	82.1	1.4	1.4	0.0	0.5	1.0	2.1	3.5	4.1	5.7
trans-caryophyllene oxide	98.2	1.3	0.9	0.0	0.6	1.1	1.8	2.5	3.0	5.5
6-hydroxycarvotanacetone	97.3	1.2	1.2	0.0	0.4	0.8	1.7	2.7	3.8	6.6
methyl ether coahuilensol	73.2	1.1	1.4	0.0	0.0	0.4	1.7	2.8	3.7	8.7
α-cadinol	92.9	0.7	0.7	0.0	0.2	0.4	0.8	1.5	2.4	3.1
isopiperitenon	94.6	0.6	0.4	0.0	0.4	0.5	0.7	1.1	1.4	3.1
β-elemene	81.3	0.6	0.6	0.0	0.2	0.4	0.7	1.4	1.7	3.0
veratrole	69.6	0.5	0.7	0.0	0.0	0.2	0.7	1.4	1.9	3.8
spathulenol	91.1	0.4	0.2	0.0	0.2	0.3	0.5	0.6	0.6	1.1
(e)-jasmone	78.6	0.4	0.3	0.0	0.1	0.3	0.5	0.8	0.9	1.9
globulol	92.9	0.3	0.2	0.0	0.2	0.3	0.4	0.6	0.7	1.0
cis-calamenene	89.3	0.3	0.2	0.0	0.1	0.2	0.3	0.6	0.7	1.0
fitone	83.0	0.3	0.3	0.0	0.1	0.2	0.4	0.7	0.9	1.2
(z)-β-farnesene	77.7	0.3	0.2	0.0	0.1	0.2	0.4	0.5	0.6	0.9
3-octanol acetate	66.1	0.3	0.3	0.0	0.0	0.2	0.4	0.7	0.7	1.1
α-terpineol	83.9	0.2	0.2	0.0	0.1	0.2	0.3	0.5	0.6	0.7
1,10-diepi-cubenol	77.7	0.2	0.2	0.0	0.1	0.2	0.3	0.4	0.5	1.2
δ-terpineol	71.4	0.2	0.2	0.0	0.0	0.2	0.3	0.4	0.5	0.7
τ-cadinol	68.8	0.2	0.3	0.0	0.0	0.1	0.3	0.4	0.6	2.4
terpinen-4-ol	68.8	0.2	0.3	0.0	0.0	0.2	0.3	0.6	0.9	1.4
germacra-4(15),5,10(14) -trien-1α-ol	67.9	0.2	0.2	0.0	0.0	0.1	0.2	0.4	0.7	0.7
torreyol	66.1	0.2	0.2	0.0	0.0	0.1	0.2	0.4	0.5	0.9

**Table 6:** Anti-*Candida* activities of the 82 EO samples and pure PO and PIP; the antifungal activity tests were carried out three times, and the average values were taken as the MICs. All standard deviation values were below 2%.

EO ID	MIC mg·mL^−1^		EO ID	MIC mg·mL^−1^		EO ID	MIC mg·mL^−1^		EO ID	MIC mg·mL^−1^	
	24 h	48 h		24 h	48 h		24 h	48 h		24 h	48 h
EO002	3.12	3.12	EO033	3.12	6.24	EO068	3.12	3.12	EO091	3.12	3.12
EO003	3.12	6.24	EO034	3.12	3.12	EO069	3.12	3.12	EO092	6.24	6.24
EO004	12.48	12.48	EO035	1.56	1.56	EO070	1.56	3.12	EO093	3.12	3.12
EO007	3.12	6.24	EO037	0.78	3.12	EO072	1.56	3.12	EO094	1.56	1.56
EO012	6.24	12.48	EO038	1.56	3.12	EO073	0.78	1.56	EO095	1.56	3.12
EO013	3.12	3.12	EO039	3.12	3.12	EO074	3.12	3.12	EO096	6.24	6.24
EO014	6.24	12.48	EO040	3.12	6.24	EO075	6.24	6.24	EO098	1.56	1.56
EO015	6.24	12.48	EO046	3.12	6.24	EO076	12.48	12.48	EO099	1.56	3.12
EO019	6.24	6.24	EO047	1.56	3.12	EO077	6.24	*na* ^1^	EO100	1.56	3.12
EO020	6.24	6.24	EO048	1.56	3.12	EO078	3.12	6.24	EO101	3.12	6.24
EO021	6.24	6.24	EO049	1.56	3.12	EO079	3.12	6.24	EO102	6.24	12.48
EO022	3.12	3.12	EO050	1.56	3.12	EO080	3.12	6.24	EO103	12.48	12.48
EO023	1.56	3.12	EO051	3.12	6.24	EO081	6.24	6.24	EO104	3.12	6.24
EO024	1.56	1.56	EO055	0.78	1.56	EO082	12.48	12.48	EO105	1.56	3.12
EO025	3.12	3.12	EO056	3.12	3.12	EO083	6.24	6.24	EO107	3.12	6.24
EO026	3.12	3.12	EO057	1.56	3.12	EO084	1.56	3.12	EO108	0.39	0.78
EO028	1.56	1.56	EO058	0.78	6.24	EO086	6.24	6.24	EO109	0.78	1.56
EO029	1.56	3.12	EO059	3.12	6.24	EO087	6.24	*na*	EO110	12.48	12.48
EO030	3.12	6.24	EO064	0.78	1.56	EO088	6.24	6.24	EO111	6.24	12.48
EO031	3.12	3.12	EO065	0.78	1.56	EO089	1.56	3.12	PO ^2^	12.48	12.48
EO032	1.56	3.12	EO066	1.56	3.12	EO090	3.12	3.12	PIP ^3^	6.24	12.48

^1^ *na* = non-active; ^2^ PO = piperitenone oxide isolated from MSEO; ^3^ PIP = lab-synthesized piperitenone.

**Table 7:** Summary of EO sampling with fractionated or continuous distillations (HD or SD) from either fresh or dried MS plant materials.

Fractionated Extraction		Continued Extraction		Complementary Continued Extraction to 24 h	
Interval Times	Duration (Hours)	Interval Times	Duration (Hours)	Interval Times	Duration (Hours)
0–1	1	0–1	1	1–24	23
1–2	1	0–2	2	2–24	22
2–3	1	0–3	3	3–24	21
3–6	3	0–6	6	6–24	18
6–24	18	0–24	24	-	-

## Supplementary Materials

The following supporting information can be downloaded at: https://www.mdpi.com/article/10.3390/molecules28196934/s1.
