# Supplementary material for: Chemical Composition and Anti-Candida Activity of Mentha suaveolens Ehrh. Essential Oils Obtained by Different Distillation Processes
Source: Molecules. 2023 Oct 4;28(19):6934. doi: 10.3390/molecules28196934 (PMC10574099; doi:10.3390/molecules28196934)
Supplement: Supplementary file 1 [file molecules-28-06934-s001.zip › molecules-2599319-supplementary.pdf]

Chemical Composition Investigation and Anti-*Candida* Activity of *Mentha suaveolens* Ehrh. Essential Oils Obtained by Continuous and Fractionated Hydrodistillation and Steam Distillation

Vanja Tadić, Mijat Božović, Filippo Sapienza, Roberta Astolfi, Milan Mladenović, Maria Cristina Zaka, Fabiana Del Bove, Francesca Borzacchi, Caterina Fraschetti, Caterina Rossi, Silvia Vertuani, Anna Baldisserotto, Stefano Manfredini and Rino Ragno

Supplementary Material

Tables:

Table S1. Share % of total (up to 24h) yields for each continued extraction.

	1st harvest				2nd harvest			
h[1]	fc-SD[2]	dc-SD[3]	fc-HD[4]	dc-HD[5]	fc-SD	dc-SD	fc-HD	dc-HD
1	26.4915	38.628	14.6985	34.333	15.5085	36.961	27.7723	43.906
2	26.4466	35.825	29.6103	42.139	43.3441	37.117	25.2706	42.188
3	41.5148	28.537	14.4229	44.585	41.8964	26.584	31.4445	47.133
6	33.3333	46.283	18.5218	46.597	49.3941	41.656	35.5808	48.391
24	100.000	100.000	100.000	100.000	100.000	100.000	100.000	100.000

[1] extraction duration; [2] fc-SD = continuous steam distillation on fresh plant; [3] dc-SD = continuous steam distillation on dried plant; [4] fc-HD = continuous hydrodistillation on fresh plant; [5] dc-HD = continuous hydrodistillation on dried plant.

Table S2. Share % of total yields over extraction time for the fractionated SDs and HDs on the fresh and dried plant.

	1st harvest				2nd harvest			
h[1]	ff-SD[2]	df-SD[3]	ff-HD[4]	df-HD[5]	ff-SD	df-SD	ff-HD	df-HD
0-1	15.6915	61.4409	52.4297	83.3489	21.4115	35.4934	43.1048	55.5271
0-2	40.8344	69.8663	73.6670	88.8827	45.9733	43.4064	63.8588	71.8449
0-3	55.8557	71.9192	83.3616	91.9925	59.1020	52.8609	75.1298	76.5939
0-6	86.0368	83.6293	98.7700	94.3486	80.2329	68.1115	85.2230	89.5976
0-24	100.000	100.000	100.000	100.000	100.000	100.000	100.000	100.000

[1] extraction hour/interval; [2] ff-SD = fractionated steam distillation on fresh plant; [3] df-SD = fractionated steam distillation on dried plant; [4] ff-HD = fractionated hydrodistillation on fresh plant; [5] df-HD = fractionated hydrodistillation on dried plant.

Figures:

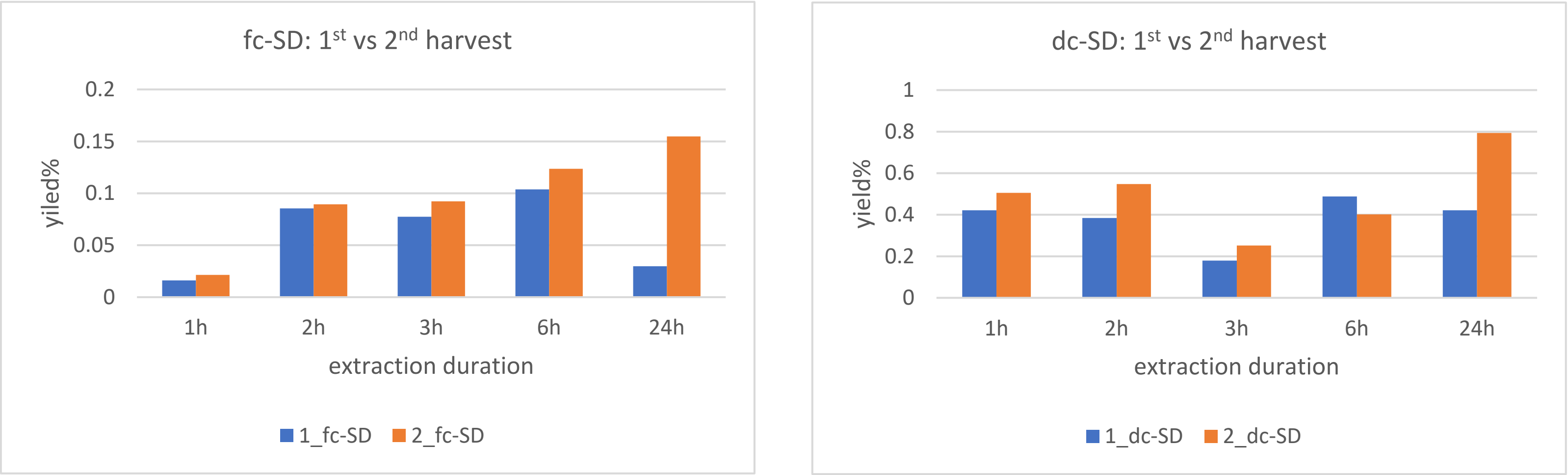

Figure S1. Yields% for continued steam distillation: fresh (left) and dried (right) material, comparation between 1st and 2nd harvest.

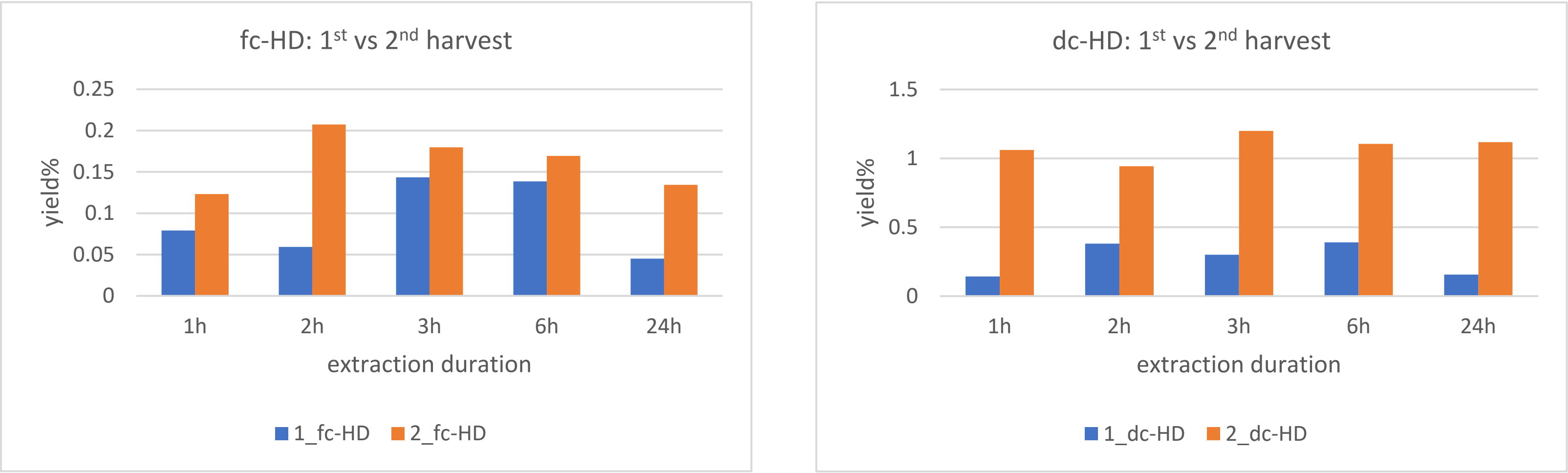

Figure S2. Yields% for continued hydrodistillation: fresh (left) and dried (right) material, comparation between 1st and 2nd harvest.

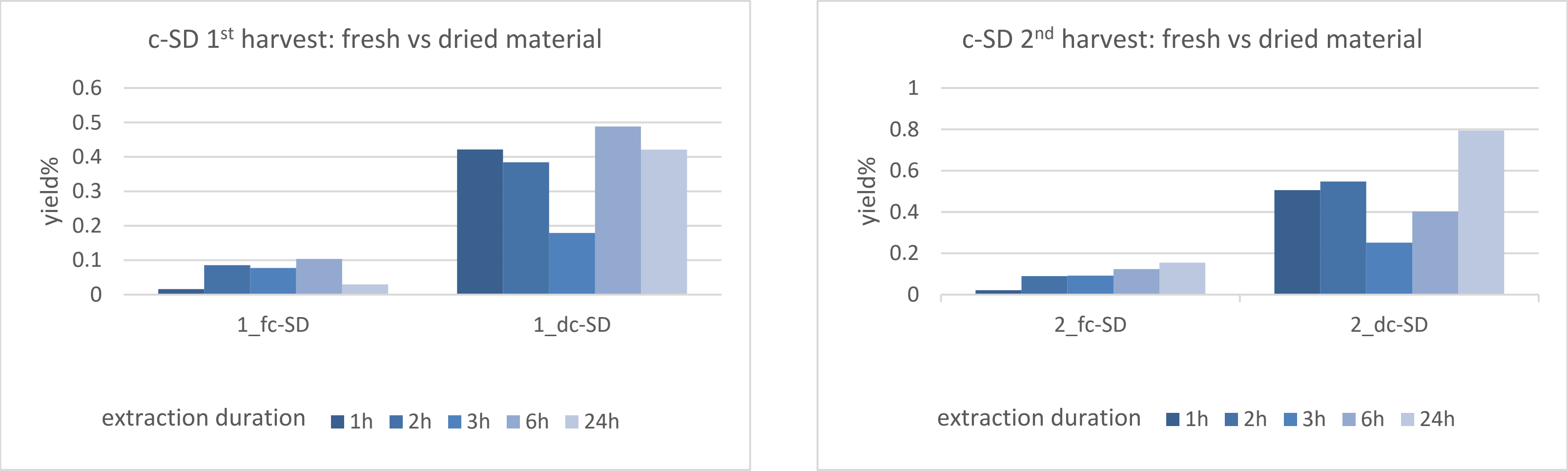

Figure S3. Yield comparation of each extraction between fresh and dried materials: continued steam distillation, 1st (left) and 2nd (right) harvest.

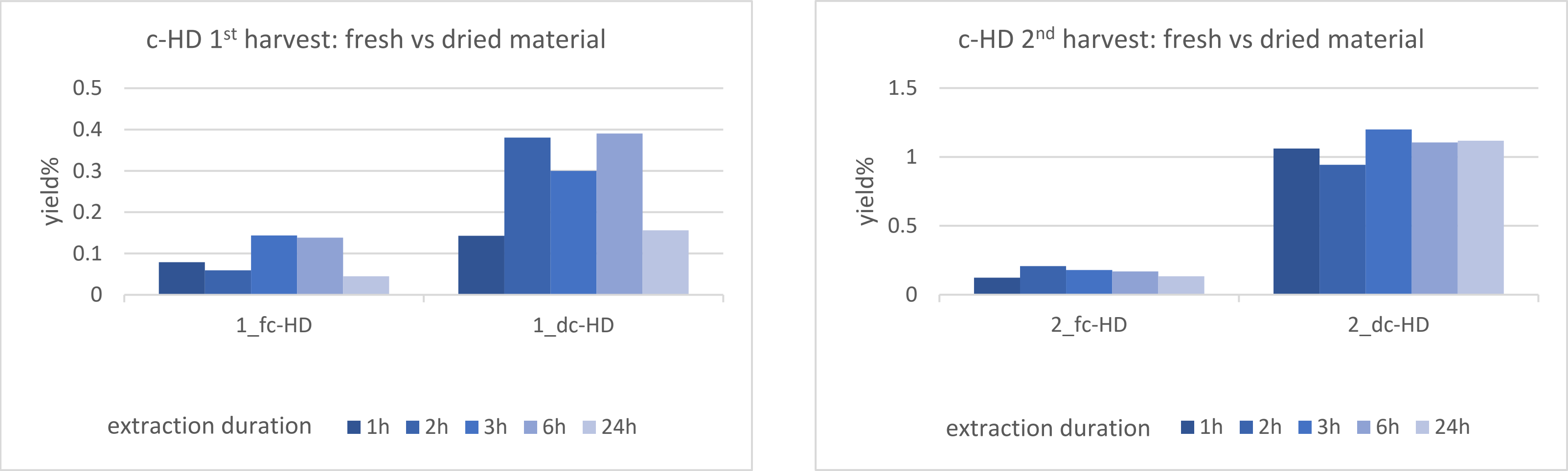

Figure S4. Yield comparation of each extraction between fresh and dried materials: continued hydrodistillation, 1st (left) and 2nd (right) harvest.

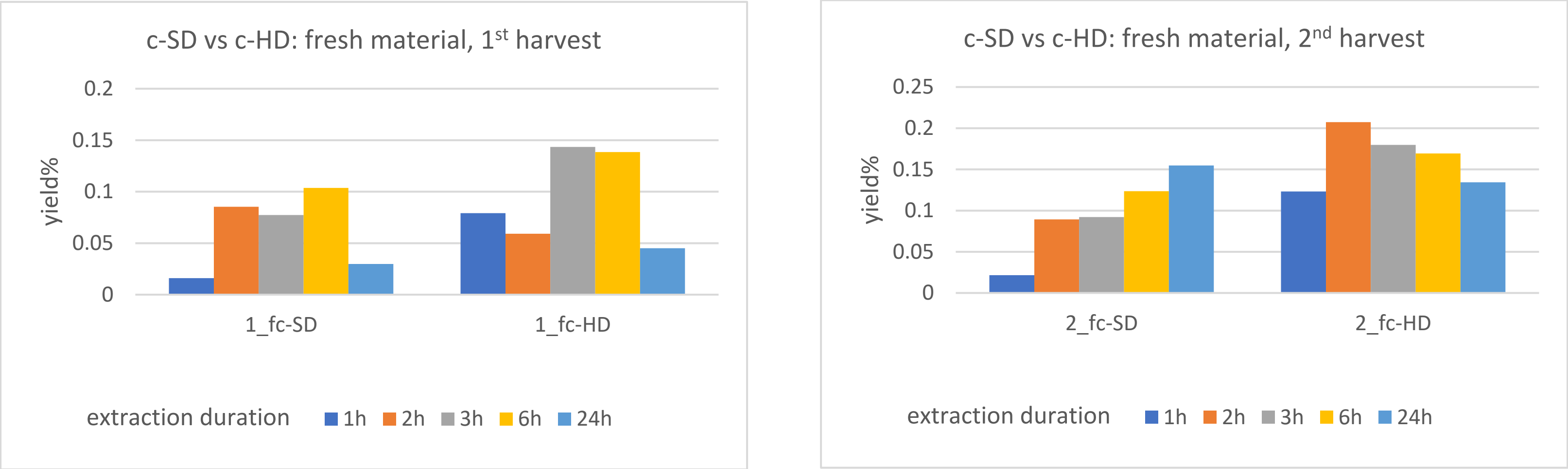

Figure S5. Comparation of the yields between continued steam and hydrodistillations: 1st (left) and 2nd (right) harvest, fresh material.

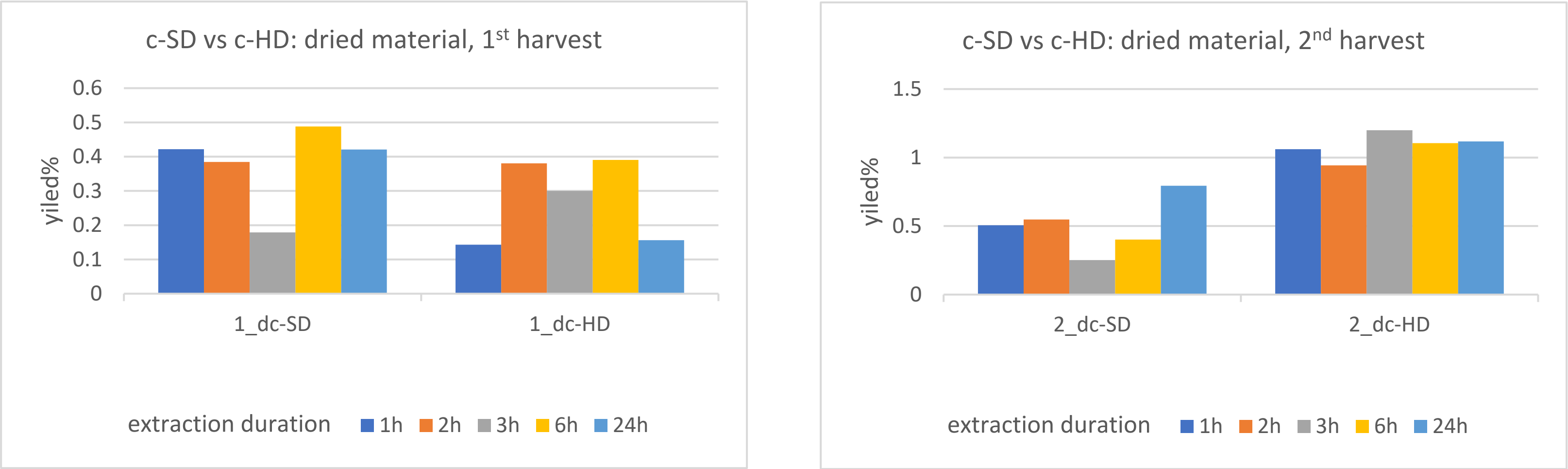

Figure S6. Comparation of the yields between continued steam and hydrodistillations: 1st (left) and 2nd (right) harvest, dried material.

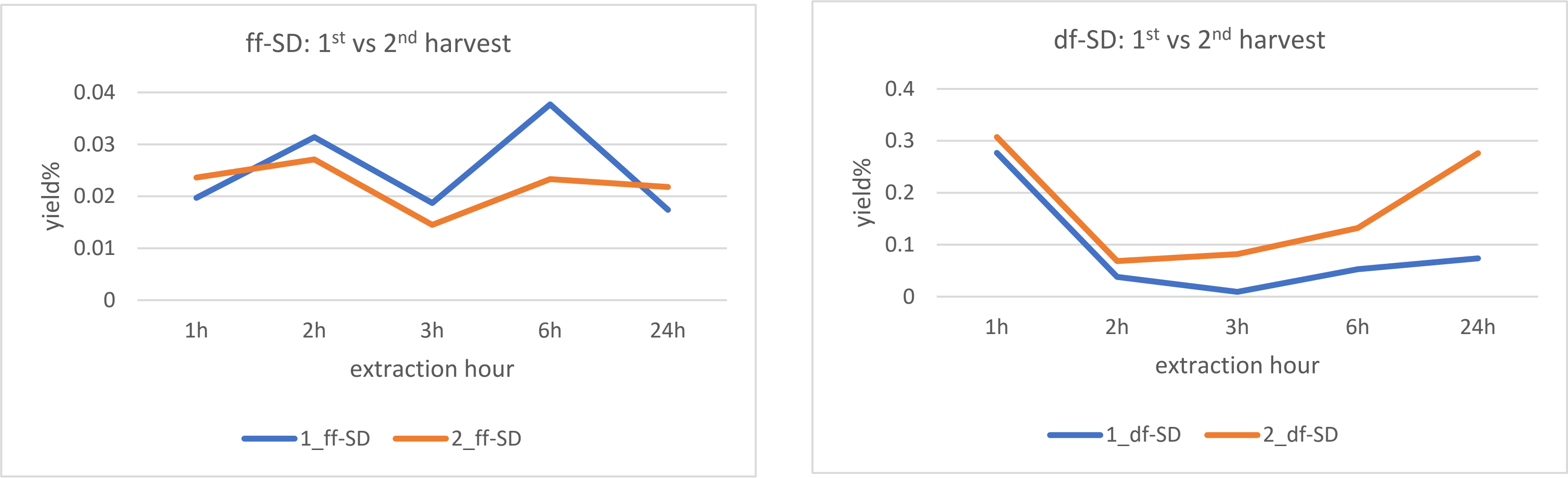

Figure S7. Comparation of the yields over the time between 1st and 2nd harvest: fractionated steam distillation, fresh (left) and dried (right) material.

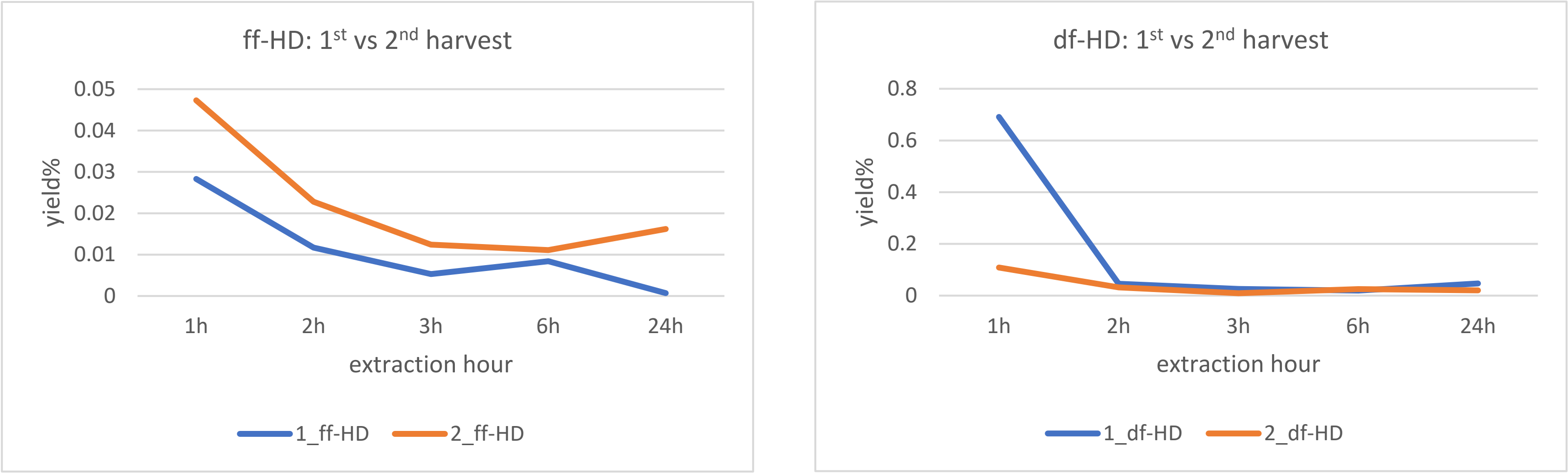

Figure S8. Comparation of the yields over the time between 1st and 2nd harvest: fractionated hydrodistillation, fresh (left) and dried (right) material.

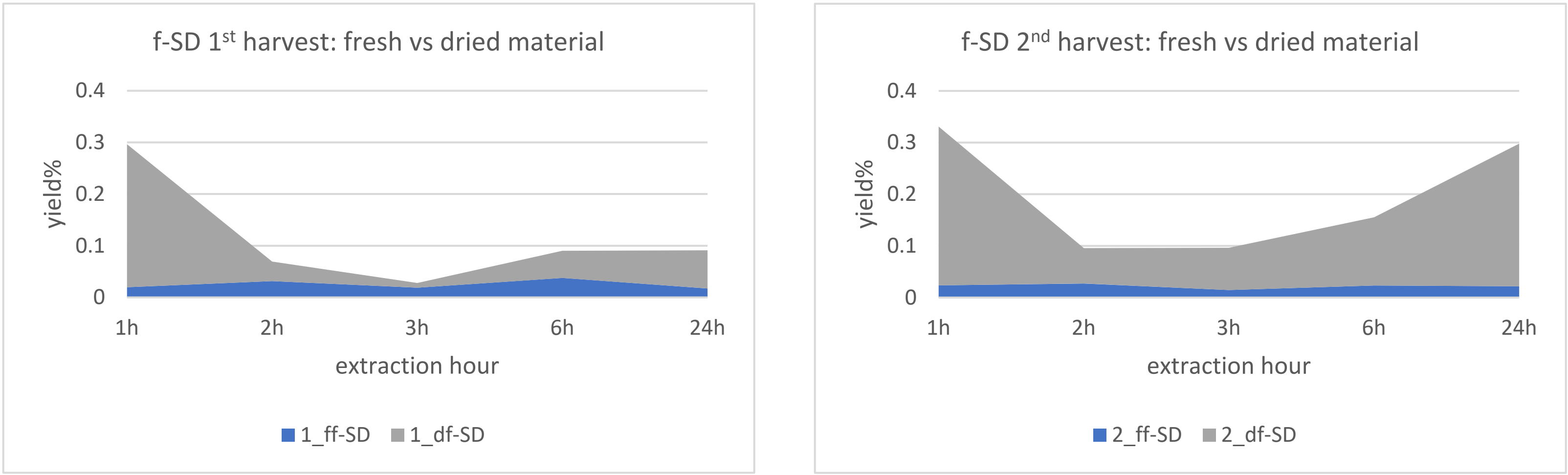

Figure S9. Comparation of the yields over the time between fresh and dried materials: fractionated steam distillation, 1st (left) and 2nd (right) harvest.

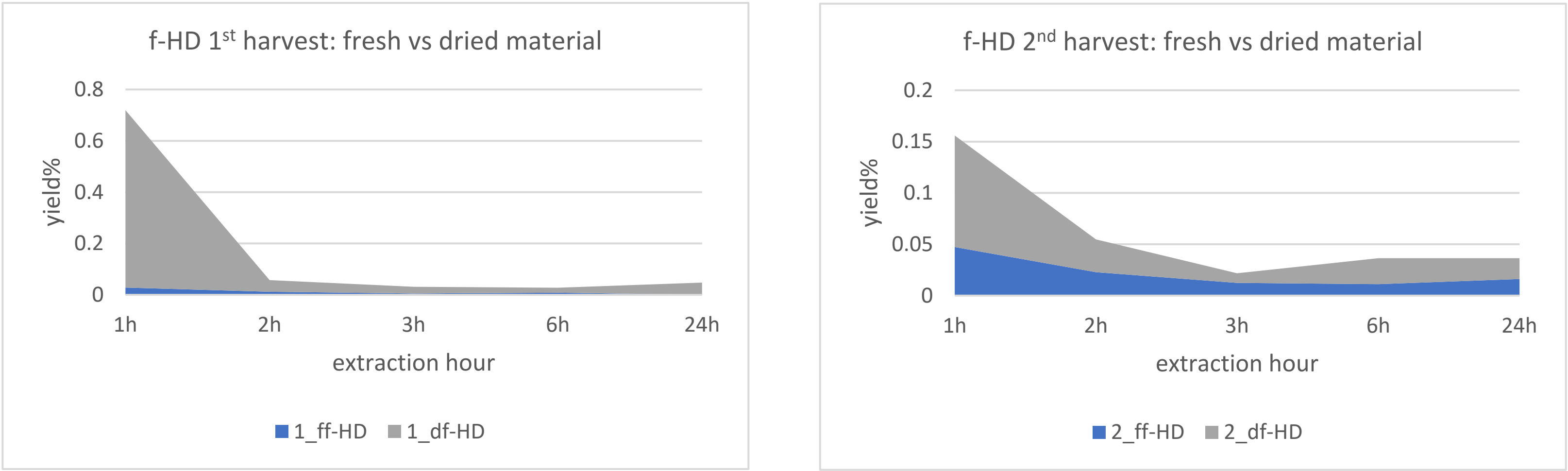

Figure S10. Comparation of the yields over the time between fresh and dried materials: fractionated hydrodistillation, 1st (left) and 2nd (right) harvest.

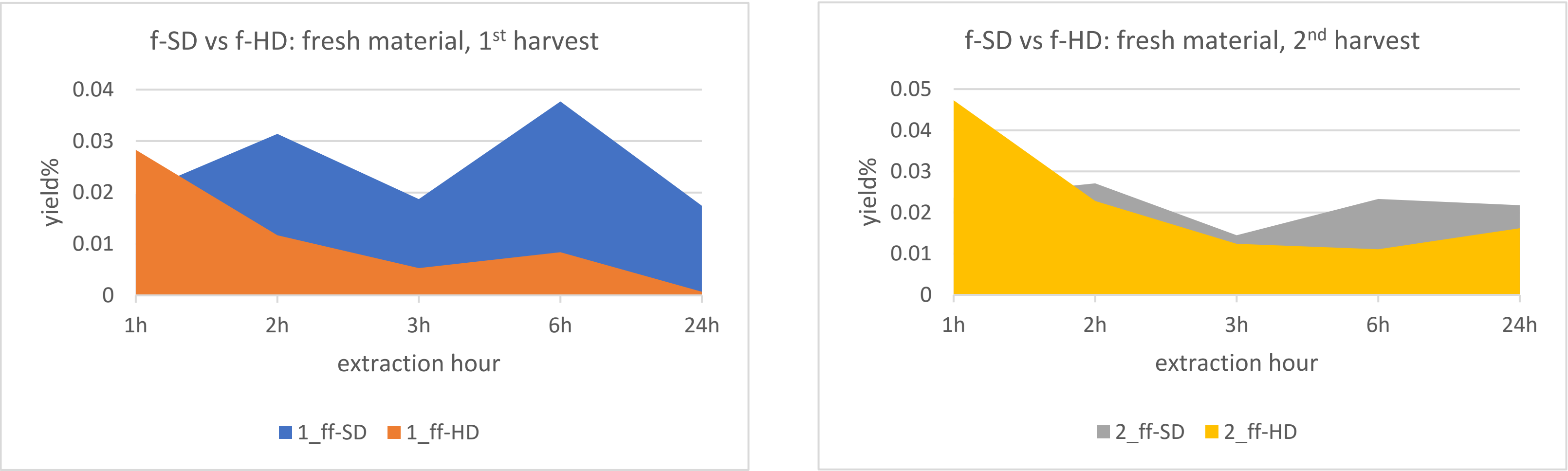

Figure S11. Comparation of the yields over the time between fractionated steam and hydrodistillations: 1st (left) and 2nd (right) harvest, fresh material.

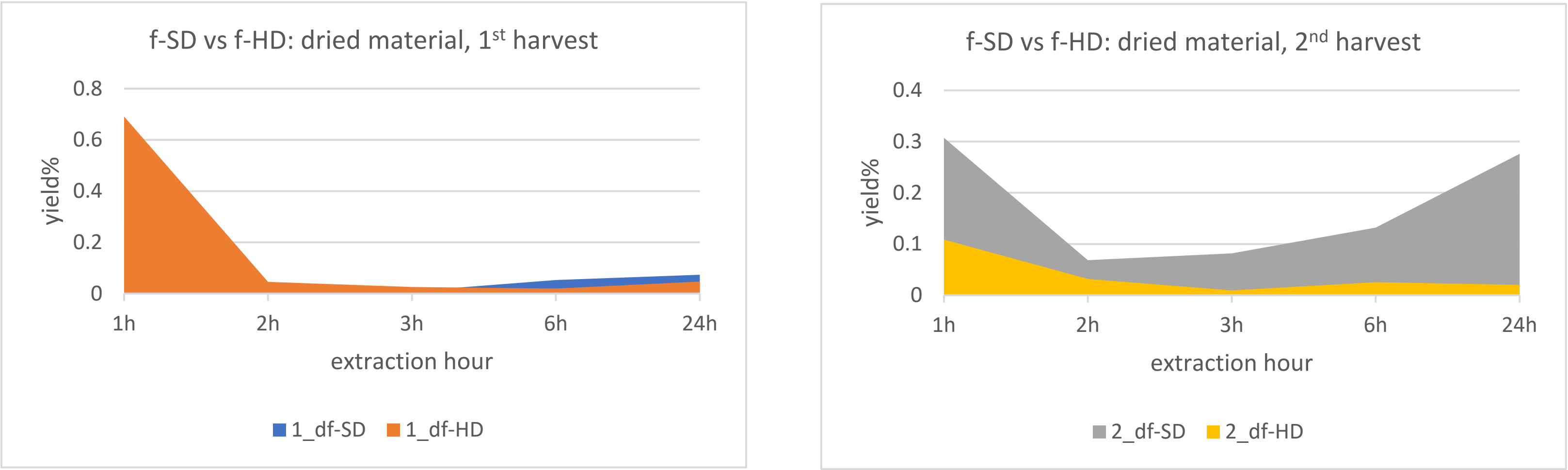

Figure S12. Comparation of the yields over the time between fractionated steam and hydrodistillations: 1st (left) and 2nd (right) harvest, dried material.
